# Central Nervous System Inflammation and Infection during Early, Nonaccelerated Simian-Human Immunodeficiency Virus Infection in Rhesus Macaques

**DOI:** 10.1128/JVI.00222-18

**Published:** 2018-05-14

**Authors:** Denise C. Hsu, Piyanate Sunyakumthorn, Matthew Wegner, Alexandra Schuetz, Decha Silsorn, Jacob D. Estes, Claire Deleage, Khamis Tomusange, Samir K. Lakhashe, Ruth M. Ruprecht, Eric Lombardini, Rawiwan Im-Erbsin, Yanin Kuncharin, Yuwadee Phuang-Ngern, Dutsadee Inthawong, Weerawan Chuenarom, Robin Burke, Merlin L. Robb, Lishomwa C. Ndhlovu, Jintanat Ananworanich, Victor Valcour, Robert J. O'Connell, Serena Spudich, Nelson L. Michael, Sandhya Vasan

**Affiliations:** a U.S. Military HIV Research Program, Department of Retrovirology, AFRIMS, Bangkok, Thailand; b Henry M. Jackson Foundation for the Advancement of Military Medicine, Bethesda, Maryland, USA; c Department of Veterinary Medicine, Armed Forces Research Institute of Medical Sciences, Bangkok, Thailand; d AIDS and Cancer Virus Program, Frederick National Laboratory for Cancer Research, Leidos Biomedical Research, Inc., Frederick, Maryland, USA; e Texas Biomedical Research Institute, San Antonio, Texas, USA; f Southwest National Primate Research Center, San Antonio, Texas, USA; g U.S. Military HIV Research Program, Walter Reed Army Institute of Research, Silver Spring, Maryland, USA; h University of Hawaii, Honolulu, Hawaii, USA; i University of Amsterdam, Amsterdam, The Netherlands; j University of California, San Francisco, California, USA; k Yale University, New Haven, Connecticut, USA; Icahn School of Medicine at Mount Sinai

**Keywords:** HIV, SHIV, early infection, neuropathology

## Abstract

Studies utilizing highly pathogenic simian immunodeficiency virus (SIV) and simian-human immunodeficiency virus (SHIV) have largely focused on the immunopathology of the central nervous system (CNS) during end-stage neurological AIDS and SIV encephalitis. However, this may not model pathophysiology in earlier stages of infection. In this nonaccelerated SHIV model, plasma SHIV RNA levels and peripheral blood and colonic CD4^+^ T cell counts mirrored early human immunodeficiency virus (HIV) infection in humans. At 12 weeks postinfection, cerebrospinal fluid (CSF) detection of SHIV RNA and elevations in IP-10 and MCP-1 reflected a discrete neurovirologic process. Immunohistochemical staining revealed a diffuse, low-level CD3^+^ CD4^−^ cellular infiltrate in the brain parenchyma without a concomitant increase in CD68/CD163^+^ monocytes, macrophages, and activated microglial cells. Rare SHIV-infected cells in the brain parenchyma and meninges were identified by RNAScope *in situ* hybridization. In the meninges, there was also a trend toward increased CD4^+^ infiltration in SHIV-infected animals but no differences in CD68/CD163^+^ cells between SHIV-infected and uninfected control animals. These data suggest that in a model that closely recapitulates human disease, CNS inflammation and SHIV in CSF are predominantly mediated by T cell-mediated processes during early infection in both brain parenchyma and meninges. Because SHIV expresses an HIV rather than SIV envelope, this model could inform studies to understand potential HIV cure strategies targeting the HIV envelope.

**IMPORTANCE** Animal models of the neurologic effects of HIV are needed because brain pathology is difficult to assess in humans. Many current models focus on the effects of late-stage disease utilizing SIV. In the era of antiretroviral therapy, manifestations of late-stage HIV are less common. Furthermore, new interventions, such as monoclonal antibodies and therapeutic vaccinations, target HIV envelope. We therefore describe a new model of central nervous system involvement in rhesus macaques infected with SHIV expressing HIV envelope in earlier, less aggressive stages of disease. Here, we demonstrate that SHIV mimics the early clinical course in humans and that early neurologic inflammation is characterized by predominantly T cell-mediated inflammation accompanied by SHIV infection in the brain and meninges. This model can be utilized to assess the effect of novel therapies targeted to HIV envelope on reducing brain inflammation before end-stage disease.

## INTRODUCTION

Early research on the effects of human immunodeficiency virus (HIV) on the central nervous system (CNS) focused on HIV-associated dementia (HAD), often occurring in late-stage HIV infection in the setting of low peripheral CD4^+^ T cell counts and high plasma viremia and characterized by HIV infection of macrophages and microglia (1, 2). Nonhuman primate models utilizing simian immunodeficiency virus (SIV) have replicated and further characterized these clinical and immunologic findings, especially within the brain parenchyma, as it is difficult to access in humans. SIV-infected rhesus macaques demonstrated motor and cognitive impairment (3), and SIV RNA was detectable within the cerebrospinal fluid (CSF) and brain parenchyma as early as 7 days postinfection (dpi), accompanied by early macrophage infiltration into the brain and immune activation (4–8). In a more accelerated SIV infection model in pigtail macaques, SIV RNA and DNA was detected early (within 4 days postinoculation for SIV RNA) within both brain tissue and CSF, accompanied by macrophage infiltration and activation within the brain (9–12). Collectively, these models have established monocytes/macrophages as the primary cells responsible for HIV-related pathologies in the brain.

Because HIV encephalitis is less common in the era of antiretroviral therapy (ART), clinical neurologic studies have expanded to encompass the broader spectrum of HIV-associated neurocognitive disorders (HAND), which include asymptomatic neurocognitive impairment, HIV-associated mild neurocognitive disorder, and HAD. Unfortunately, cognitive defects, motor defects, and neuropathy can persist despite effective ART, although treatment earlier in the course of HIV infection may reduce their frequency and severity (13–16). Viral replication can be compartmentalized in the CNS early in HIV infection (17, 18), and studies of CSF indicate that early HIV infection is associated with intrathecal immune activation, brain inflammation, and neuronal dysfunction on magnetic resonance spectroscopy (19, 20).

The more recently proposed potential therapies for HIV cure are mainly intended for use in earlier stages of infection, well before the onset of sequelae of advanced HIV infection. Many such therapies, including therapeutic vaccines and monoclonal antibodies, are targeted against the HIV envelope, necessitating simian-human immunodeficiency virus (SHIV) expressing HIV envelope to model their effects on the CNS in rhesus macaques. Harbison et al. have reported the effects of SHIV-SF162p3 on the CNS but focused on end-stage encephalitis in an AIDS model (21). However, clinical studies indicate that CNS involvement during early infection is a pathologically distinct process. The description of genetically compartmentalized viruses involved in this early persistent CNS replication that were adapted to replicate in CD4^+^ T cells in CSF from recently infected individuals (18) differs from findings of a macrophage-dominated CNS process in macaque models that cause more aggressive disease during early infection.

This study characterizes the effect of a subtype C SHIV-1157ipd3N4 infection on the CSF and brain parenchyma of rhesus macaques in a nonaccelerated model that appears to mimic the clinical course of early HIV infection in humans.

## RESULTS

### SHIV-1157ipd3N4 mimics early HIV infection.

**FIG 1:**
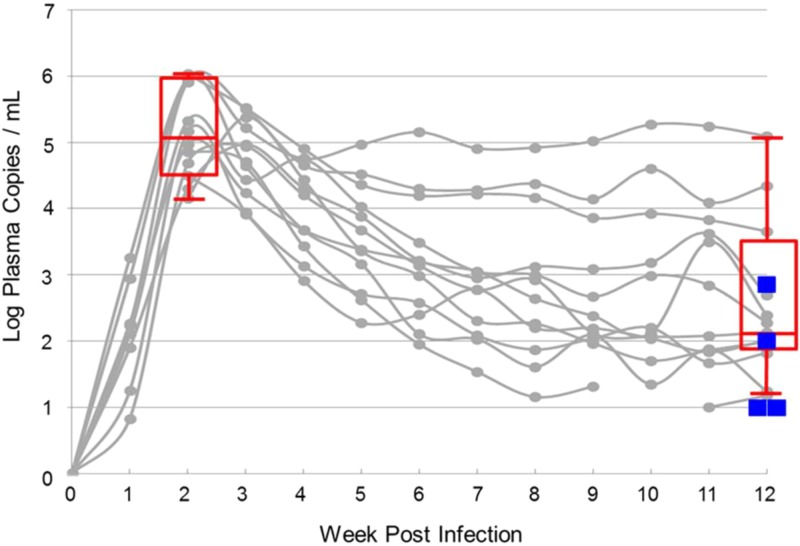
SHIV RNA in plasma and CSF during early infection. Gray circles represent individual plasma viremia after intrarectal (*n* = 9) or intravaginal (*n* = 3) SHIV inoculation at week 0. Red box-and-whisker plots depict medians, interquartile ranges, and ranges of plasma SHIV levels at weeks 2 and 12 postinfection. Blue squares represent CSF SHIV RNA levels in the four animals with detectable SHIV RNA in the CSF at week 12 postinfection, corresponding to the four animals with the highest plasma viremia at the same time point.

Twelve Indian-origin rhesus macaques were prescreened to exclude the restrictive Mamu A*01, B*08, and B*17 alleles and inoculated with a single dose of SHIV-1157ipd3N4 (an R5-tropic mucosally transmissible virus encoding an HIV subtype C *env* derived from a Zambian infant [22]) either intrarectally (9 males) or intravaginally (3 females). All animals were monitored for 12 weeks postinfection for weekly plasma viremia and CD4^+^ T cell count, as well as colonic biopsy specimens at 3 weeks postinfection (W3) and 12 weeks postinfection (W12). The kinetics of plasma SHIV RNA quantified by reverse transcription-PCR (RT-PCR) mimicked early HIV infection in humans, with mean peak viremia of 5.3 log_10_ copies/ml (range, 4.2 to 5.9 log_10_ copies/ml) and W12 set point viremia at 4.1 log_10_ copies/ml (range, 1.2 to 5.1 log_10_ copies/ml). There was no effect of SHIV inoculation titer on peak or set point viremia (data not shown). SHIV RNA was detectable in the CSF of the four animals with the highest W12 plasma viral load (mean, 1.7 log_10_ copies/ml; range, 1.0 to 2.7 log_10_ copies/ml) (Fig. 1). The W12 CSF/serum albumin ratio was <5 × 10^−3^ in all 12 animals (mean, 3.0 × 10^−3^; range, 0.6 × 10^−3^ to 3.2 × 10^−3^), consistent with an intact blood-brain barrier (23, 24).

**FIG 2:**
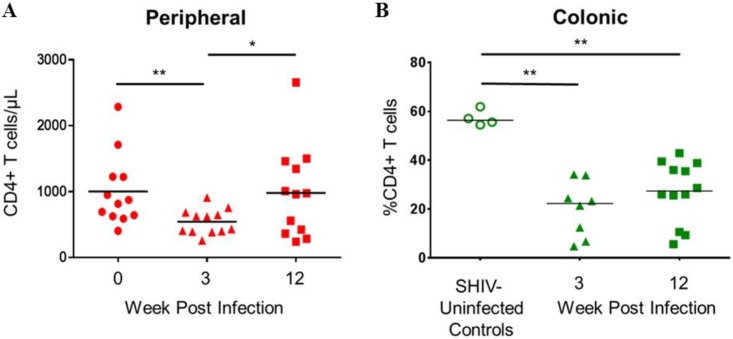
Peripheral and colonic CD4^+^ T cell depletion occurs early in infection. (A) Longitudinal peripheral blood CD4^+^ T cell counts at preinfection baseline, acute infection (W3), and set point (W12). (B) CD4^+^ T cell percentages from colonic biopsy specimens at acute infection (W3) and set point (W12) with respect to uninfected controls. Horizontal lines represent mean values. **, *P* ≤ 0.005; *, *P* ≤ 0.05. *P* values were calculated with Wilcoxon matched-pairs signed rank tests (A) or Mann-Whitney tests (B).

Peripheral blood CD4^+^ T cell depletion occurred at W3 (preinfection versus W3, 1,003 versus 543 cells/mm^3^; *P* value of 0.0010) but rebounded by W12 (W3 versus W12, 543 versus 982 cells/mm^3^; *P* value of 0.0161) (Fig. 2A). Similarly, the frequency of colonic CD4^+^ T cells decreased at W3 compared to that of SHIV-uninfected controls (20.1% versus 57.8%; *P* value of 0.0040) but did not decline further by W12 (W3 versus W12, 20.1% versus 27.1%; *P* value of >0.05) (Fig. 2B). Over the course of infection, plasma SHIV RNA was inversely correlated with peripheral (*r* = −0.44; *P* value of 0.03) and colonic (*r* = −0.62; *P* value of 0.005) CD4^+^ T cells.

### Soluble markers of inflammation in CSF are distinct from those in plasma.

**FIG 3:**
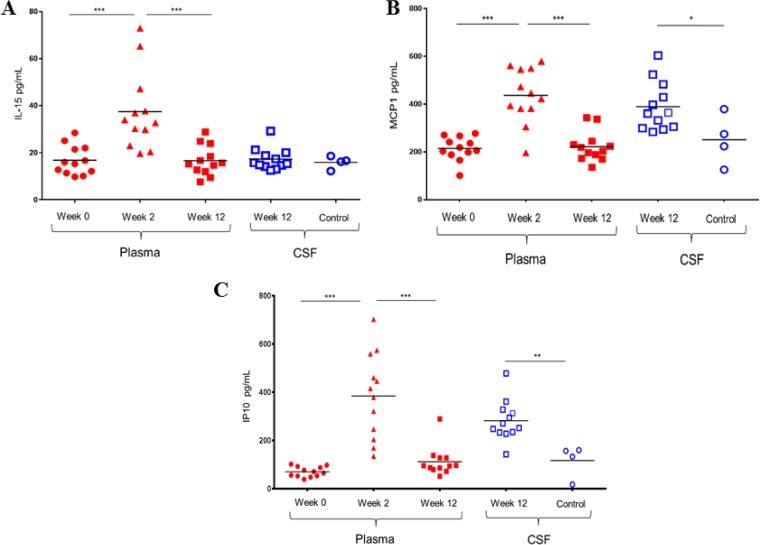
Soluble markers of inflammation in CSF are distinct from those in plasma. (A to C) Longitudinal depiction of soluble IL-15 (A), MCP-1 (B), and IP-10 (C) in plasma prior to SHIV infection at week 0 (solid circles), at peak viremia 2 weeks postinfection (solid triangles), and at set point viremia 12 weeks postinfection (solid squares). Horizontal lines represent mean values. Levels in CSF 12 weeks postinfection (open squares) were compared to those in CSF from healthy uninfected control macaques. ***, *P* ≤ 0.0005; **, *P* ≤ 0.005; *, *P* ≤ 0.05. *P* values were calculated with Wilcoxon matched-pairs signed rank tests (plasma) or Mann-Whitney tests (CSF).

At 2 weeks postinfection, plasma interleukin-15 (IL-15), monocyte chemoattractant protein-1 (MCP-1/CCL2), and induced protein 10 (IP-10) were significantly elevated compared to the preinfection baseline (W0 versus W2, 16.7 versus 37.5 pg/ml [*P* value of 0.0005], 215 versus 436 pg/ml [*P* value of 0.0005], and 70 versus 384 pg/ml [*P* value of 0.0005], respectively) and normalized by W12 (W2 versus W12, 37.5 versus 16.6 pg/ml [*P* value of 0.0005], 436 versus 222 pg/ml [*P* value of 0.0005], and 384 versus 112 pg/ml [*P* value of 0.0005], respectively). While there was no elevation of IL-15 in the W12 CSF compared to the level for uninfected control CSF, MCP-1 and IP-10 levels in W12 CSF were significantly elevated compared to those of control CSF (390 versus 251 pg/ml [*P* value of 0.0396] and 282 versus 117 pg/ml [*P* value of 0.0044], respectively) (Fig. 3). Neopterin was similarly elevated in plasma at W2 over preinfection baseline (18.3 versus 6.3 pg/ml; *P* value of <0.0001) and normalized by W12 and was not different in W12 CSF versus control CSF. All other soluble factors assessed by the nonhuman primate multiplex cytokine kit (Merck) were either undetectable or unchanged among time points or groups. CD4^+^ T cell frequencies in sigmoid biopsy specimens at weeks 0, 2, and 12 correlated inversely with plasma levels of IP-10 (*r* = −0.48; *P* value of 0.04), IL-15 (*r* = −0.58; *P* value of 0.01), and MCP-1 (*r* = −0.46; *P* value of 0.04), suggesting a link between mucosal CD4^+^ T cell depletion and systemic immune activation.

### Predominant T cell inflammatory infiltrate in brain parenchyma.

**FIG 4:**
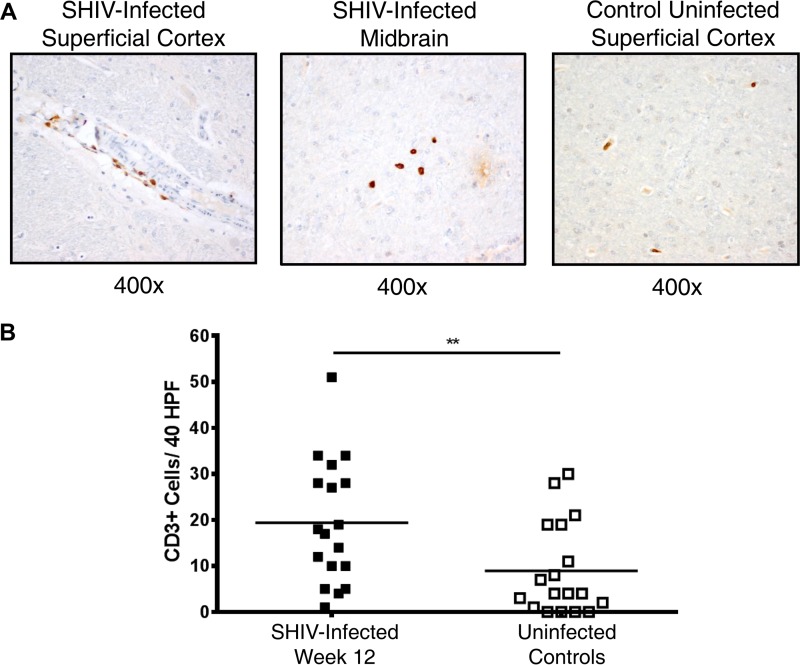
CD3^+^ T cell infiltrate into brain parenchyma in early SHIV infection. (A) Representative images of CD3^+^ T cell staining in the superficial cortex and midbrain of SHIV-infected animals and an uninfected control. CD3^+^ T cells (brown) are depicted pavementing along the endothelium of a blood vessel in the superficial cortex (top left) in an SHIV-infected animal and in the midbrain parenchyma of a different SHIV-infected animal (top middle). In contrast, CD3^+^ T cell staining in uninfected control animals was limited to intravascular spaces in all regions (top right) without evidence of endothelial pavementing. (B) Quantification of CD3^+^ T cell counts per 40 HPF in midbrain, basal ganglia, and superficial cortex in the six SHIV-infected animals with the highest viremia 12 weeks postinfection (solid squares) versus uninfected controls (open squares). Horizontal lines represent mean values. **, *P* < 0.01; calculated with the Mann-Whitney test.

**FIG 5:**
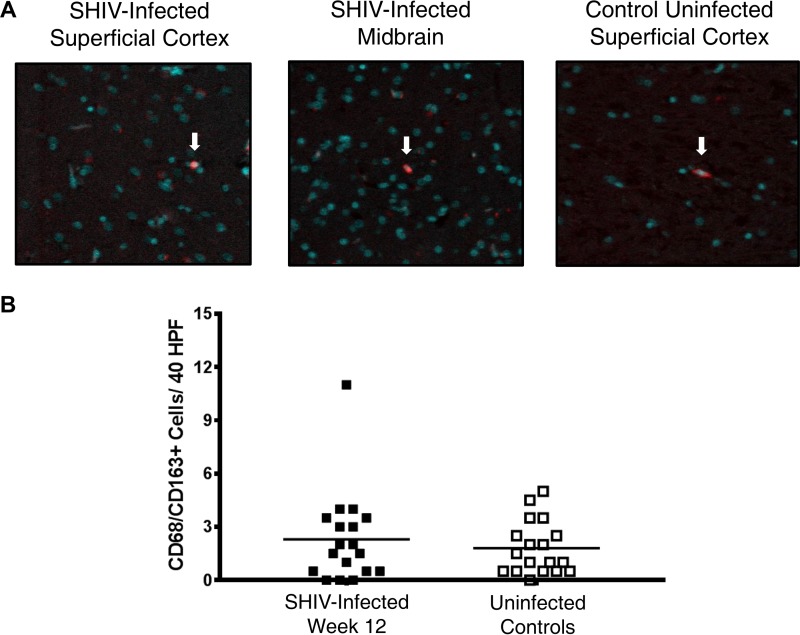
No significant increase in CD68/CD163^+^ cell infiltrate in brain parenchyma in early SHIV infection. (A) Representative images of CD68/CD163^+^ staining in the superficial cortex and midbrain of SHIV-infected animals and an uninfected control. White arrows indicate cells positive for CD68/CD163 (red). DAPI staining of nuclei is depicted in aqua. (B) Quantification of CD68/CD163^+^ cell counts per 40 HPF in midbrain, basal ganglia, and superficial cortex in the six SHIV-infected animals with the highest viremia 12 weeks postinfection (solid squares) versus uninfected controls (open squares). Horizontal lines represent mean values. *P* > 0.05; calculated with the Mann-Whitney test.

Staining on formalin-fixed, paraffin-embedded brain tissues for CD3^+^ T cells, CD4^+^ cells, and CD68/CD163^+^ cells (monocytes, macrophages, and/or activated microglial cells) was conducted in the superficial cortex, basal ganglia, and midbrain 12 weeks postinfection in the six animals with the highest plasma viremia and compared with the same tissues from six healthy SHIV-uninfected control animals. There was evidence of pavementing, or leukocyte adherence to capillary endothelium, of CD3^+^ T cells along the vascular endothelium and CD3^+^ T cell migration into the brain parenchyma in SHIV-infected animals but not in uninfected controls, where CD3^+^ T cells were localized in the intravascular spaces (Fig. 4A). Across the superficial cortex, midbrain, and basal ganglia, SHIV-infected animals demonstrated a mild increase in CD3^+^ T cell infiltrate compared to the level for uninfected control animals (19.4 ± 13.4 cells/40 high-power fields [HPF] versus 8.9 ± 10.0 cells/40 HPF; *P* value of 0.0084), which was not localized to any particular region (Fig. 4B). However, there was no evidence of an increase in CD4^+^ infiltrate versus uninfected controls in the frontal cortex, midbrain, or basal ganglia despite positive staining in meninges (0.7 ± 1.0 cells/40 HPF versus 1.1 ± 1.3 cells/40 HPF; *P* value of >0.05). Similarly, there was no significant increase in CD68/CD163^+^ cells in these regions compared to levels for uninfected controls (2.3 ± 2.6 cells/40 HPF versus 1.8 ± 1.5 cells/40 HPF; *P* value of >0.05) (Fig. 5).

**FIG 6:**
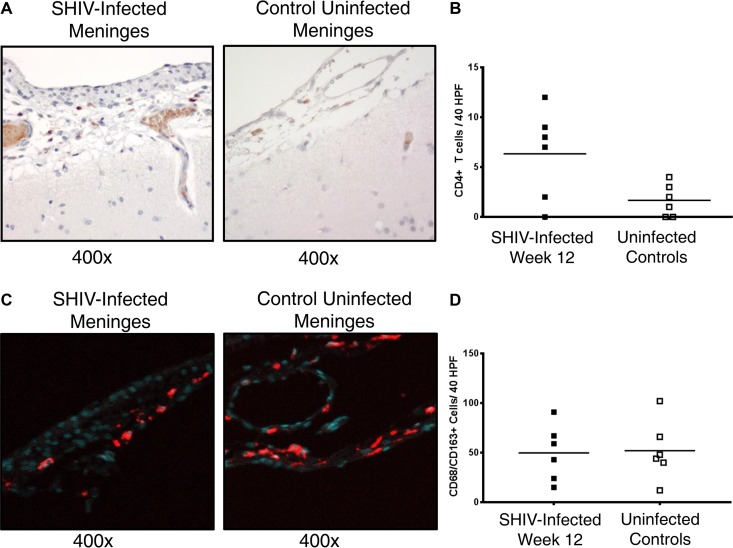
CD4^+^ cell infiltrate into meninges of SHIV-infected macaques without concomitant increase of CD168/CD163^+^ cell infiltrate. (A) Representative immunohistochemical staining of meninges revealing a mild CD4^+^ cell infiltrate (dark brown) into meninges 12 weeks after SHIV infection (left) but not in uninfected controls (right). Light brown represents nonspecific staining inside meningeal vessels, whereas dark brown represents extravascular CD4^+^ cells within the meningeal space. (B) Quantification of CD4^+^ cell counts per 40 HPF in meninges in SHIV-infected macaques (solid squares) versus uninfected controls (open squares), depicting increased CD4^+^ cell infiltrate in four of six animals relative to healthy controls, three of whom had detectable SHIV RNA in CSF. Horizontal lines represent mean values. *P* > 0.05; calculated with the Mann-Whitney test. (C) Representative immunofluorescent staining of meninges in an SHIV-infected animal and uninfected control, with CD68/CD163^+^ cells depicted in red and DAPI-stained nuclei depicted in aqua. (D) Quantification of CD68/CD163^+^ cell counts per 40 HPF in meninges in SHIV-infected macaques (solid squares) versus uninfected controls (open squares), showing no significant differences between groups. Horizontal lines represent mean values. *P* > 0.05; calculated with the Mann-Whitney test.

To determine a possible source of SHIV RNA in the CSF, we examined the CD4^+^ and CD68/CD163^+^ cells in the meninges and choroid plexus of the same infected and control animals. CD4^+^ cell infiltration was increased in the meninges in four out of six SHIV-infected animals versus controls, although differences in the level of CD4^+^ cells in meninges between all six SIV-infected animals and six healthy controls did not reach statistical significance (6.3 ± 4.5 cells/40 HPF versus 1.6 ± 1.6 cells/40 HPF; *P* value of 0.1017) (Fig. 6). Three of the four animals with meningeal CD4^+^ cell infiltrate had detectable SHIV RNA in CSF. CD68/CD163^+^ cells were identified in variable numbers in the meninges from SHIV-infected animals and uninfected controls (49.8 ± 28.3 cells/40 HPF versus 52 ± 30.1 cells/40 HPF; *P* value of >0.05) without a similar trend toward a difference between groups. Low numbers of CD4^+^ cells (1.2 ± 2.0 cells/40 HPF versus 0.4 ± 0.5 cells/40 HPF; *P* value of >0.05) and CD68/CD163^+^ cells (1.6 ± 1.1 cells/40 HPF versus 0.7 ± 0.8 cells/40 HPF; *P* value of >0.05) were detected in the choroid plexus of SHIV-infected animals or uninfected controls, respectively.

### SHIV-infected cells are detectable in the superficial cortex and meninges 12 weeks postinfection.

**FIG 7:**
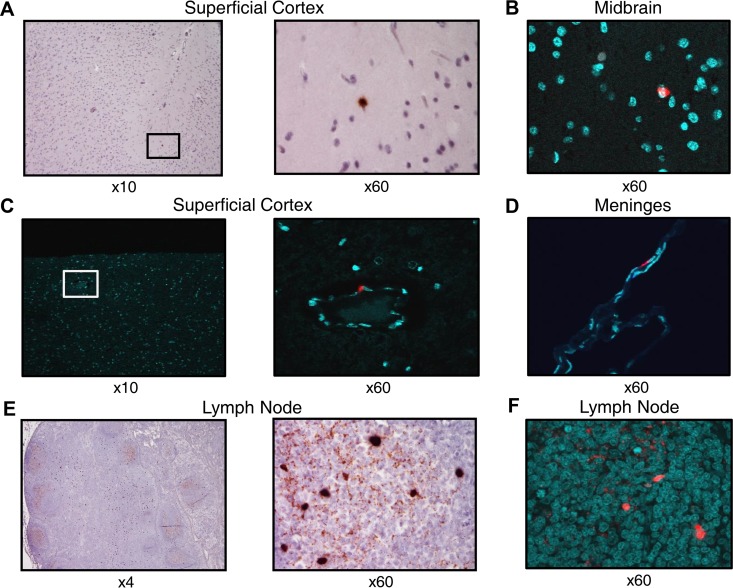
SHIV RNA^+^ cells in superficial cortex and meninges 12 weeks postinfection. RNAScope staining of tissue using light (A and E) or confocal (B, C, D, and F) microscopy. (A) SHIV RNA^+^ cells (brown) in superficial cortex near a cortical sulcus (10×, left) and at higher magnification (60×, right). (B) SHIV RNA^+^ cells (red) in midbrain parenchyma with DAPI costaining of cell nuclei (aqua). (C) SHIV RNA^+^ cells (red) colocated with cells along the endothelium of a cortical blood vessel with DAPI costaining of cell nuclei (aqua). (D) SHIV RNA^+^ cells (red) within the meninges with DAPI costaining of cell nuclei (aqua). (E and F) SHIV RNA^+^ cells and virions within mesenteric lymph node follicles as positive controls (brown and red).

Formalin-fixed paraffin-embedded sections of brain parenchyma and meninges from the six animals with highest plasma viremia at week 12 were selected and hybridized with probes to SIVmac239 using RNAScope technology (25). Detection of chromogenic signal by light microscopy or fluorescent signal by confocal microscopy revealed the presence of rare but clearly demonstrable SHIV RNA-positive cells in both the brain parenchyma and meninges in five out of six animals (Fig. 7), including 1 animal with undetectable SHIV RNA in the CSF, while all healthy SHIV-uninfected control samples were unilaterally negative by RNAScope *in situ* hybridization. Congruent with the CD3^+^ T-cell distribution pattern, SHIV RNA^+^ cells were also identified along the endothelial vasculature in the brain.

### SHIV-1157ipd3N4 replicates in monocyte-derived macrophages.

**FIG 8:**
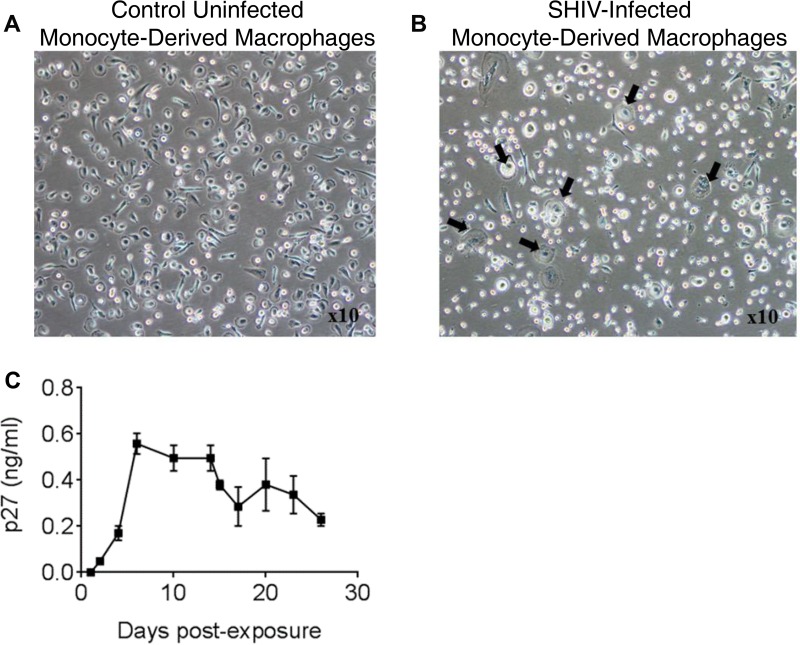
SHIV-1157ipd3N4 replication in MDMs. MDMs were exposed to SHIV-1157ipd3N4 at an MOI of 0.03 (see Materials and Methods). (A) Uninfected control MDMs; (B) syncytium formation in SHIV-1157ipd3N4-exposed MDMs. Black arrows indicate multinucleated giant cells; magnification, ×10. (C) p27 levels in supernatants collected from SHIV-1157ipd3N4-exposed MDMs. A blood pack of an anonymous human donor was used to prepare the cells used in this experiment. Data are representative of two independent experiments; error bars represent means ± standard deviations.

Because macrophages are primary cellular targets for HIV infection (2, 26, 27), we sought to confirm the ability of SHIV-1157ipd3N4 to replicate in MDMs. An uninfected control culture was maintained throughout (Fig. 8A). In cultures exposed to SHIV-1157ipd3N4, multinucleated giant cells were visible under the light microscope 7 days postexposure (Fig. 8B). Viral p27 was first detected on day 2 and peaked on day 6 (Fig. 8C). Collectively, these results demonstrate that exposure of MDMs to SHIV-1157ipd3N4 resulted in productive infection and virus-induced cytopathic effect.

## DISCUSSION

Here, we report the early CNS effects associated with SHIV-1157ipd3N4 infection, which has a less aggressive course than many strains of SIV generally used to model CNS infection (2–4, 28). The kinetics of plasma viral load of SHIV-1157ipd3N4 infection simulated early HIV infection in humans with the caveats of lower peak and set point viremia and higher rates of spontaneous virologic control (29). Early CD4^+^ T cell depletion in the peripheral blood and colon shortly after peak viremia consistently reversed by 12 weeks postinfection, mimicking CD4^+^ T cell dynamics during early HIV infection (29–32). The SHIV RNA level in CSF 12 weeks postinfection was 2 logs lower than that in plasma and was also accompanied by elevations of the proinflammatory chemokines IP-10 and MCP-1 in the CSF, also consistent with observations in humans early in the course of HIV infection (19). Taken together, these findings demonstrate that subtype C SHIV-1157ipd3N4 models the early course of HIV infection in the CNS and other tissue compartments in many aspects (33).

The mild CD3^+^ CD4^−^ infiltrate at week 12 postinfection was presumably a CD8^+^ T cell infiltrate which was not localized to any specific brain region, as levels were similar among basal ganglia, superficial cortex, and midbrain, although it remains possible that there are differences in other regions not yet studied.

Sturdevant et al. have described four states of the relationship between virus in the CSF/CNS and blood during early infection, progressing from equilibration of viruses between blood and CSF to clonal amplification and eventual persistent replication within the CNS (18). In our model, the two-log difference between blood and CSF levels of SHIV RNA with an intact blood-brain barrier is consistent with the first two stages of this process. In this study, RNAScope staining revealed the presence of rare but clearly detectable SHIV-infected cells in five out of six animals distributed across both meninges and brain parenchyma, consistent with other reports (34).

Some studies have postulated that early entry of HIV into the CNS is mediated by systemic inflammation causing disruption to the blood-brain barrier, thereby facilitating cellular trafficking to the CNS and subsequent neuroinflammation (5, 6, 18). However, it is also possible that SHIV-infected cells in the CNS primarily arise from an enhanced migration of activated T cells across an intact blood-brain barrier. Thus, the subtle cognitive and motor defects in early HIV infection may be a result of diffuse, low-level inflammation within the brain driven by cytokines/chemokines and CD8^+^ T cells rather than a direct effect of HIV itself, as CD8^+^ T cells have been shown to correlate with early CNS dysfunction in the SIV model (35).

Alternatively, recent studies have described the presence of functional lymphatic vessels lining the dural sinuses which absorb fluid from the subarachnoid space, carry fluid and cells from the CSF, and connect to the deep anterior cervical lymph nodes (36–39). Given that four out of six animals had a meningeal CD4^+^ T cell infiltrate, that SHIV RNA-expressing cells were visualized in the meninges 12 weeks postinfection, and that proinflammatory chemokines were elevated in CSF, it is possible that early brain infection occurs via transport of virus to the meninges via meningeal blood vessels or lymphatics.

The lack of increased CD68/CD163^+^ cells in the SHIV-infected brain parenchyma, meninges, and choroid plexus does not refute the well-established role of monocytes/macrophages in trafficking HIV into the brain (1, 2, 4–9). *In vitro* experiments also provided evidence against the hypothesis that the lack of increase in monocytes/macrophages was due to the inability of SHIV-1157ipd3N4 to infect these cells. Rather, this model suggests that HIV entry into the CNS compartment is biphasic, with a mild, early T cell-mediated infiltrate followed by a more pathogenic monocyte-dominated infiltrate later in the course of disease. Similarly, because the presence of viral RNA in the brain does not necessarily equate to productive infection, these findings do not implicate that these earliest foci of infection are those that establish latent reservoirs. Potential limitations of this study include the lower virulence of SHIV-1157ipd3N4 compared with that of HIV and the clonal nature of the SHIV-1157ipd3N4 inoculum, making a differential selection of quasispecies in the blood versus CNS during initial seeding unlikely. However, this affords the opportunity to monitor viral evolution over time in future longitudinal studies.

With increasing emphasis on treatment as prevention, initiation of therapy in early infection, and functional HIV cure, there is an increasing focus on administering interventions early in the course of HIV infection. This SHIV model reflects early human neuropathogenesis more closely than accelerated SIV models and confers an added advantage over SIV models due to the expression of HIV envelope. These features will enable investigations into the early CNS effects of therapeutic vaccines and monoclonal antibodies directed against the HIV envelope.

## MATERIALS AND METHODS

### Study design.

This study was designed to test the hypothesis that less pathogenic, R5-tropic SHIV can mimic the viral dynamics and mild CNS inflammation characteristic of the earliest stages of clinical HIV infection. Nine male and three female adult Indian-origin rhesus macaques (Macaca mulatta) at the AAALAC International-accredited Armed Forces Research Institute of Medical Science (AFRIMS; Bangkok, Thailand), Department of Veterinary Medicine, primate colony were enrolled into a protocol approved by the Institutional Animal Care and Use Committee (IACUC). Macaques with major histocompatibility complex (MHC) alleles restrictive for SIV infection and Mamu A*01, B*08, and B*17 were excluded for enrollment as described below and prescreened for baseline clinical stability via complete blood count, serum chemistry, and general physical examination. On study week 0, males were inoculated intrarectally with 1 ml SHIV-1157ipd3N4 diluted at 1:10 (*n* = 3), 1:25 (*n* = 3), 1:50 (*n* = 2), or 1:100 (*n* = 1), while females were inoculated intravaginally with SHIV-1157ipd3N4 diluted 1:5. Females were clinically monitored to avoid inoculation during menses, but no hormonal treatment was administered prior to infection. Postinfection, macaques were clinically observed at least three times daily and underwent weekly phlebotomy. At week 3 postinfection, animals underwent colonic biopsy specimen under isoflurane anesthesia. Animals were humanely euthanized at week 12, at which time blood, CSF, and tissues from brain, meninges, lymph nodes, and colon were obtained.

### Ethics statement.

All care and use of animals were in compliance with the *Guide for the Care and Use of Laboratory Animals* (40). Animal use protocols were approved by the IACUC and Biosafety Review Committee at AFRIMS, Bangkok, Thailand, an AAALAC International-accredited facility. SHIV-infected samples described in the manuscript were obtained from macaques under protocol PN13-07. CSF, serum, and tissues from brain, meninges, lymph nodes, and colon from four healthy control animals and brain samples from six healthy control animals from the same AFRIMS primate colony were obtained from a separate IACUC-approved protocol, PN12-07.

All animal research was conducted in compliance with Thai laws, the Animal Welfare Act, and all applicable U.S. Department of Agriculture, Office of Laboratory Animal Welfare, and U.S. Department of Defense guidelines. All animal research adhered to the *Guide for the Care and Use of Laboratory Animals* (40).

After SHIV inoculation, monkeys were individually housed in standard stainless steel cages with a minimum floor space of 6.4 square feet and exposed to ambient environmental conditions inside an animal biosafety level 2 (ABSL-2) containment laboratory. Monkeys were fed daily with commercially prepared Old World primate extruded feed and supplemented with fresh fruit or vegetables four times per week. Fresh chlorinated water (5 to 10 ppm) was provided *ad libitum* via automatic water valves. Cages were cleaned daily and sanitized biweekly. Under the behavioral and environmental enrichment program, food enrichment, structural enrichment, and animal training were provided to promote the psychological and physical well-being of animals in the facility. The animals were observed three times daily by trained veterinary staff. The sample collection was performed under anesthesia using ketamine hydrochloride. At the study endpoint, animals were euthanized by ketamine hydrochloride injection followed by barbiturate in accordance with the *Guidelines for the Euthanasia of Animals* (41).

### Sample processing and Mamu typing.

To isolate peripheral blood mononuclear cells (PBMCs), blood from EDTA tubes was centrifuged and PBMCs were isolated with Histopaque (Sigma) density gradient prior to washing and freezing at below −150°C until use. Plasma, serum, and CSF were centrifuged and supernatant was frozen at −80°C. DNA for MHC analyses was isolated using a QIAamp DNA minikit (50) (Qiagen) according to the manufacturer's protocol. To identify certain MHC class I alleles, Mamu-A*01, Mamu-B*08, and Mamu-B*17, DNA was extracted from 200 μl EDTA-anticoagulated blood using the QIAamp DNA minikit (50) (Qiagen) according to the manufacturer's protocol. The genomic DNA was genotyped for the MHC class I alleles by PCR amplification with Platinum Taq DNA polymerase, PCR optimization buffer B, sequence-specific primers, and internal control primers as previously described (42).

### SHIV RT-PCR.

Total RNA was isolated from EDTA plasma using a QIAamp viral RNA minikit (50) (Qiagen). RNA from macaque plasma or viral standards underwent quantitative PCR (qPCR) amplifications with TaqMan fast virus 1-step master mix (Applied Biosystems) and pSGAG23 probe (5′-6-carboxyfluorescein [6FAM]-CTTCCTCAGTKTGTTTCACTTTCTCTTCTGCG-6-carboxytetramethylrhodamine [TAMRA]-3′) (Applied Biosystems). The following forward and reverse primers were used for qPCR amplification: 5′-GTCTGCGTCATCTGGTGCATTC-3′ and 5′-CACTAGKTGTCTCTGCACTATCTGTTTTG-3′ (Applied Biosystems). PCR conditions were 50°C for 5 min and 95°C for 20 s for 1 cycle each and then 95°C for 15 s at 60°C for 1 min for 45 cycles. qPCRs were carried out using a 7900 PCR system (Applied Biosystems). Results were expressed as copies/milliliter based on interpolation to the standard curve as previously described (43).

### Peripheral and colonic CD4^+^ T cell quantification.

For peripheral CD4^+^ T cell quantification from whole blood, whole EDTA blood was stained with the following monoclonal antibodies (MAbs): fluorescein isothiocyanate (FITC)-conjugated anti-CD3e (BD), allophycocyanin (APC)-conjugated anti-CD4 (BD), phycoerythrin (PE)-conjugated anti-CD8 (BD), and peridinin chlorophyll protein (PerCP)-conjugated CD45 (BD) in TruCount tubes (BD). Stained sample was lysed with fluorescence-activated cell sorting (FACS) lysing buffer (BD), and CD3/CD4/CD8/CD45^+^ cell count was analyzed by acquisition of at least 2,500 gated lymphocytes on a FACSCalibur (BD). To quantify CD4^+^ T cells from colonic mucosal mononuclear cells (MMCs), flow cytometry was performed on freshly isolated MMCs from 10 to 12 colonic biopsy pieces that were processed within 30 min of collection as previously described (33). In brief, biopsy specimens were weighed and placed in 500 ml RPMI medium containing 10% human AB serum (Gemini Bio-Product, West Sacramento, CA, USA), 1% HEPES, 1% l-glutamine (l-Glut), 0.1% gentamicin (Invitrogen, Carlsbad, CA, USA), 1% penicillin-streptomycin, and 2.5 mg/ml amphotericin B (Invitrogen). Samples were digested using 0.5 mg/ml collagenase II (Sigma, St. Louis, MO, USA) and then filtered through a cell strainer twice or thrice using a syringe with a 16-gauge blunt-end needle. After being washed twice with RPMI medium, MMC were counted and then stained using FITC-conjugated anti-CD3e, APC-conjugated anti-CD4, PerCP-conjugated anti-CD45 (BD Pharmingen, San Jose, CA, USA), and PE-conjugated-anti-CD8 (BD Biosciences, San Jose, CA, USA). Subsequently cells were resuspended in 1% formaldehyde and acquired within 24 h using a BD FACSCalibur (BD, San Jose, CA, USA) and analyzed using FlowJo software, version 9.6.4 or higher (TreeStar, Ashland, OR, USA). At least 80,000 live cells were acquired in the lymphocyte gate.

### Plasma and CSF soluble factors.

The nonhuman primate multiplex cytokine kit (Merck) was utilized to quantify the following cytokines and chemokines in plasma and CSF: granulocyte CSF (G-CSF), granulocyte-macrophage CSF (GM-CSF), gamma interferon (IFN-γ), IL-1b, IL-1ra, IL-2, IL-4, IL-5, IL-6, IL-8, IL-10, IL-12/23(p40), IL-13, IL-15, IL-17a, IL-18, MCP-1, MIP-1b, MIP-1a, SCD-40L, tumor growth factor alpha (TGF-α), tumor necrosis factor alpha (TNF-α), and vascular endothelial growth factor (VEGF). Standards or 25 μl of undiluted serum or plasma was added to wells in duplicate and processed according to the manufacturer's protocol. Data were analyzed using MILIPLEX Analyst 5.1 software (Merck) utilizing best fitting to standard curves, and results were expressed as picograms per milliliter. IFN-γ-induced protein 10 (IP-10/CXCL10) levels in plasma at weeks 0, 2, and 12 postinfection and CSF at week 12 postinfection were quantified using rhesus macaque IP-10 quantitative enzyme-linked immunosorbent assay (ELISA) kits (RayBiotech, Inc.). IP-10 standards or undiluted plasma or CSF samples were added to wells, incubated for 2.5 h at room temperature, washed four times, and incubated for 1 h at room temperature with biotinylated anti-rhesus macaque IP-10 antibody. After washing, wells were incubated with horseradish peroxidase (HRP)-conjugated streptavidin for 45 min at room temperature followed by chromogenic substrate, 3,3′,5,5′-tetramethylbenzidine (TMB) solution, for 40 min at room temperature. Absorbance at 450 nm was measured immediately after addition of stop solution. The quantity of IP-10 was interpolated from the standard curve. Results were expressed as picograms per milliliter.

### Albumin assay.

To assess the integrity of the blood-brain barrier, albumin levels were measured in serum and in CSF 12 weeks postinfection. Serum or CSF supernatant postcentrifugation was assayed in the albumin monkey ELISA kits (Abcam PLC) per the manufacturer's instructions. Results were expressed as a ratio of CSF to serum albumin times 10^−3^, where a ratio of <5 × 10^−3^ is indicative of an intact blood-brain barrier (23, 24).

### Immunohistochemistry.

Tissue samples were fixed in 10% formalin for at least 48 h, processed in an automated tissue processor (TP1020; Leica, Buffalo Grove, IL, USA), paraffin embedded, sectioned at 5 μm using a semiautomated rotary microtome (RM2245; Leica, Buffalo Grove, IL, USA), and mounted onto poly l-lysine-coated microscope slides.

For CD3 and CD4 staining, tissues on slides were deparaffinized, rehydrated, and placed into a PT-link tank (Dako, Glostrup, Denmark) filled with 3-in-1 target retrieval solution (Dako, Glostrup, Denmark) at 95°C for 20 min. Slides were allowed to cool to 65°C and then transferred to EnVision FLEX wash buffer (Dako, Glostrup, Denmark) for 20 min at room temperature. Tissue sections from SHIV-infected (*n* = 6) and control (*n* = 6) monkeys were stained with primary antibodies to CD3 (polyclonal; Dako, Glostrup, Denmark) and CD4 (clone BC/1F6; Abcam, Cambridge, UK) in antibody diluent for 30 min at room temperature. Negative-control slides were incubated with antibody diluent alone. The slides were washed and incubated with N-Histofine simple stain AP (MULTI) (Nichirei Biosciences Inc., Chuo-ku, Tokyo, Japan) for 30 min at room temperature. The slides were washed again and incubated with liquid permanent red solution (Dako, Glostrup, Denmark) for 5 min. Slides were then counterstained with Mayer's hematoxylin solution (Sigma, St. Louis, MO, USA) for 5 min, washed, dehydrated with ethanol, and cleared with xylene prior to mounting with toluene-based mounting medium (Thermo Fisher Scientific, Kalamazoo, MI, USA).

For dual CD68/CD163 costaining, after deparaffinization, antigen retrieval was performed using a pretreatment reagent kit (310020; Advanced Cell Diagnostics, Hayward, CA, USA) according to the manufacturer's instructions and blocked with 5% goat serum and 5% bovine serum albumin in Tris-buffered saline with 0.1% Tween 20 (TBS-T) for 30 min. Slides were then incubated with mouse anti-CD68 (clone KP1; Biocare Medical, Pacheco, CA, USA) and mouse anti-CD163 (clone 10D6; Leica Biosystems, Newcastle Upon Tyne, UK) for 2 h at room temperature, washed, and incubated with goat anti-mouse IgG Alexa Fluor 647 (Thermo Fisher Scientific, Rockford, IL, USA) for 2 h at room temperature. Slides were then washed, counterstained with DAPI (4,6-diamidino-2-phenylindole; Thermo Fisher Scientific), and mounted with ProLong gold antifade mountant (Thermo Fisher Scientific).

### RNAScope.

RNAScope *in situ* hybridization was performed as previously described (25). In brief, formalin-fixed, paraffin-embedded (FFPE) brain tissues on slides were deparaffinized and placed in xylene followed by absolute ethanol. Slides were then pretreated with a pretreatment reagent kit (Advanced Cell Diagnostics, Hayward, CA) according to the manufacturer's instructions. Slides were then hybridized with SIVmac239-specific probes (Advanced Cell Diagnostics, Hayward, CA) at 40°C for 2 h and amplified sequentially following the 2.0 HD detection kit–brown (Advanced Cell Diagnostics, Hayward, CA) procedure at 40°C in a HybEZ hybridization oven per the manufacturer's instructions.

For chromogenic detection, slides were incubated with a 1:1 ratio of DABa and DABb (ACD) for 10 min at room temperature, followed by counterstaining with Mayer's hematoxylin solution and mounting with toluene mounting medium (Thermo Fisher Scientific, Kalamazoo, MI, USA). Images were captured using an Olympus BX43 microscope and cellSens software. For fluorescent detection, slides were incubated with TSA plus cyanine 3.5 (PerkinElmer, Waltham, MA, USA) for 5 min at room temperature, followed by counterstaining with DAPI and mounting with ProLong gold antifade mountant (Thermo Fisher Scientific). Images were captured using an Olympus FV10i confocal microscope with a 60× phase-contrast oil-immersion objective (numeric aperture, 1.35) and applying a sequential mode to separately capture the fluorescence from the different fluorochromes at an image resolution of 512 by 512 pixels.

### Generation and infection of monocyte-derived macrophages with SHIV-1157ipd3N4.

Monocytes were isolated from human PBMCs to high purity and prepared by Ficoll gradient centrifugation by negative selection using magnetic beads and the monocyte isolation kit II (Miltenyi). To differentiate monocytes into macrophages, 1.5 × 10^7^ cells were incubated at 37°C in 5% CO_2_ for 7 days in RPMI supplemented with 10% fetal calf serum (FCS), penicillin-streptomycin (pen-strep), l-Glut, and 50 ng/ml GM-CSF. Monocyte-derived macrophages (MDMs) were exposed to SHIV-1157ipd3N4 diluted in RPMI medium supplemented with 10% FCS, antibiotics, and l-Glut at a multiplicity of infection (MOI) of 0.03 as described previously (44). The inoculum was left on the cells for about 16 h, after which the cells were washed 3 times with sterile phosphate-buffered saline (PBS), and fresh GM-CSF-free medium was added. Fractions of virus-containing culture supernatants were collected starting on day 3 and then every 2 days until 26 days postexposure. MDMs were examined by light microscopy to detect virus-associated cytopathic effects (CPE) and through quantifying p27 in culture supernatants by ELISA (ABL).

### Statistical analyses.

Statistical analysis was performed with GraphPad Prism software. Comparisons of peripheral CD4^+^ T cell counts or soluble plasma markers within animals were performed using Wilcoxon matched-pairs signed-rank tests. Comparisons of colonic CD4^+^ T cell counts or soluble CSF markers between animals were performed using the Mann-Whitney test. To account for rare cellular infiltrate, immunohistochemical quantification was conducted by counting CD3^+^ cells, CD4^+^ cells, or CD68/CD163^+^ cells per 40 HPF, and results were compared between infected and uninfected control groups using the Mann-Whitney test. All *P* values are two sided, with a *P* value of less than 0.05 considered significant. Spearman rank correlations were used to examine bivariate associations between continuous study outcomes including SHIV RNA level and CD4^+^ peripheral blood or colonic T cell count, as well as mucosal CD4^+^ T cell frequency and plasma soluble factors.
